# Effects and Environmental Features of Mountainous Urban Greenways (MUGs) on Physical Activity

**DOI:** 10.3390/ijerph18168696

**Published:** 2021-08-17

**Authors:** Ming Ma, Liang Ding, Huaiyun Kou, Shaohua Tan, Hao Long

**Affiliations:** 1 School of Architecture and Urban Planning, Chongqing University, Chongqing 400030, China; tsh@cqu.edu.cn (S.T.); Longhao@cqu.edu.cn (H.L.); 2 School of Architecture and Urban Planning, Key Laboratory of Technology for Construction of Cities in Mountain Area of Ministry of Education, Chongqing University, Chongqing 400030, China; 3 School of Architecture and Design, Zhejiang University of Technology, Hangzhou 310023, China; 4 College of Architecture and Urban Planning, Tongji University, Shanghai 200092, China; khy@tongji.edu.cn

**Keywords:** mountainous urban greenway, physical activity, environmental feature, planning and design

## Abstract

The role played by urban greenways in supporting physical activity (PA) for public health benefits has been receiving increasing attention. Most studies on this topic have been conducted in plains, whereas studies in mountainous regions have been limited as a result of the complexity of these areas. To address this knowledge gap, this article aims to analyze the effects of the environmental features for a mountainous urban greenway (MUG) on PA, leading to better support for greenway practice in mountainous areas. A volunteered geographic information (VGI) method was applied through the mobile app KEEP to collect 1314 valid records of PA including its density (total PA distance per unit area within the buffer zone) and attributes (distance, duration and speed) in the Yuzhong Peninsula, Chongqing, China. Similar to those of the studies conducted in the plains, our results showed that PA density was affected by the MUG and its surrounding environment. The key environmental features included residential density, open-space POI, the slope, the transportation-MUG (T-MUG) ratio and the recreational-MUG (R-MUG) ratio. For the PA attributes, the MUG showed stronger effects than the surrounding environment. The key environmental features included the slope and type of MUG, of which the former was negatively associated with distance and speed. The T-MUG ratio was positively related to duration and speed, while the R-MUG ratio was positively related to duration and distance. No association was found between livelihood (L-MUG) and PA. Hence, to support both the PA density and attributes, the environmental qualities for recreation and the types of R-MUGs should be given more consideration in practice.

## 1. Introduction

In developing countries, the current decline in physical activity (PA) due to rapid urbanization and motorization has become a major threat to public health. In China, overweight and obesity rates among adults are 30.1% and 11.9%, respectively. Obesity-related chronic diseases account for 86.6% of the total death toll. The regular exercise rate among adults is only 18.7% [1,2].

Regular PA is an effective pathway for preventing many types of chronic diseases and increasing the wellbeing of a person. It can reduce obesity rates among adolescents, improve the quality of life of the elderly and alleviate the impacts of long-term diseases, as well as improving mood, relieving stress and promoting social support [3,4,5,6,7]. Compared with indoor environments, PA conducted in outdoor green spaces can bring more health benefits and help shape healthy lifestyles by providing social capital and cohesion [8,9,10,11]. A cross-sectional study found PA was related to urban environmental features by examing the MVPA of 6822 adults aged 18–66 years from 14 cities in 10 countries by objective means. It indicated that different environmental settings might influence PA to various degrees globally [12]. Another internatioall study for PA intervention showed that increased access to space for PA, community-scale and street-scale urban design could be applicable to promote PA generally [13]. In East Asia, green open spaces are the main locations for outdoor exercises that could produce long-term health benefits, especially for seniors [14,15,16]. Urban green spaces are believed to have long-term and population-wide effects on healthy behaviors [17]. As a result, the promotion of PA in green open spaces is becoming an important pathway for improving public health.

As linear green spaces, urban greenways could improve public health by encouraging both recreational and transportation PA [18,19]. A greenway is a “linear open space established either along a natural corridor, such as a waterfront, stream valley, or ridgeline, or overland along a railroad right-of-way that has been converted to recreational use, a canal, a scenic road, or another route” [20]. Except for their ecological functions, urban greenways allow users to engage in a variety of activities, such as recreation, transportation and social interaction. As multi-functional linear spaces, they also serve as transportation routes connecting communities, public facilities and open spaces [21,22]. Since 2000, greenway projects have been booming in China as a result of their ecological and urban benefits. In major cities, they have been used as measures to balance urban development and natural conservation. Meanwhile, the role of urban greenways in promoting PA has been gradually receiving more attention [23,24,25].

From the perspective of environmental design, physical features and urban design qualities could affect the people’s walking behavior [26]. Studies in the urban environment and transportation proposed the 5Ds theory: people’s PA could be influenced by built environment features including design, density, diversity, destination accessibility and distance to transit [27]. As a multi-purpose linear pathway, the environmental features of an urban greenway, such as its width, length, pavement, shape and location, are reported to associate with PA [28]. The planning of amenities, such as restrooms, shelters and water fountains, is reported to affect PA duration [11,29]. In a survey of 417 users in Turkey, six factors of lighting, drinking water and restroom facilities were related to use duration, while proximity and accessibility were reported to affect the frequency of use [30]. Surrounding environmental characteristics in the macro-level, such as residential density, mixed land use and public transportation, could also affect PA. Greenways located in dense residential areas, as well as in areas with mixed land use and street networks, are more likely to support PA. Greenway intervention could affect both moderate-to-vigorous physical activity (MVPA) and overall physical activity (MET-minutes/week), whereas it decreases when the greenway is at an increasing distance to the residence, indicating a dose-response effect between them [31]. Connections to transit points, squares and parks also help to improve the frequencies of PA. Some studies have reported that the surrounding environments of greenways play important roles in encouraging PA because of accessibility and proximity. A study in China proposed that providing greenways close to communities, key public services and other urban clusters could increase their use [32]. Having these close affects the usage of greenways because residents prefer to use greenways near their homes or with ease of access [20,23]. Other studies have emphasized the network attributes, which are closely associated with the connectivity of greenways, that attract people by connecting urban clusters and green spaces [25].

Many studies have provided useful insights into the effects and environmental features of urban greenways on PA, but there are few studies on the greenways in mountainous urban areas, so a knowledge gap still exists. In China, nearly two-thirds of all the towns and cities are located in mountainous areas. Many greenway projects are currently underway in these areas. Most have been planned and designed following the principles in the plains, leading to negative consequences. To help with this issue, our study sought to answer the following questions: What are the effects of mountainous urban greenways (MUGs) on PA? What are their key environmental features? Are these features the same as those in the cities located in the plains? By answering these questions, we hope to provide a basis for the improvement of PA in MUGs.

MUGs are quite different from the urban greenways in the plains in three aspects. First, MUGs have obvious three-dimensional features because of the terrain. They are less dependent on road networks and can form independent, non-motorized transportation systems that create pedestrian-only areas with few intersecting roads [33]. Second, MUGs usually are rich in environmental features such as trails, ramps, stairways and trestle that provide flexible adaptation to the terrain, so they are more complex than the greenways in the plains and create various spaces, such as parks, platforms and squares, located between them and buildings. Third, the walking radii of MUGs are usually smaller than those of the greenways in the plains because of the sloped terrain [34]. Also, few types of PA are supported by MUGs because jogging, running and cycling are more difficult to perform. Because of these differences, solutions applicable in the plains may not be appropriate for MUGs.

MUGs may produce more health benefits than the greenways in the plains because mountain walking is a more intense PA [35,36]. Naturally, the average PA of residents in mountainous areas is higher [37]. For the same distance, mountain hiking consumes an equivalent amount of calories as does jogging in the plains, thus helping to prevent obesity-related diseases, such as stroke, coronary heart disease and osteoporosis, as well as increasing the concentration of high-density lipoproteins in the body [37,38]. Climbing stairways is also a therapy for back pain. A long-term study of 1000 men showed that walking up or down 20–34 floors per week (i.e., 3–5 floors per day) reduces the chance of stroke by 29% [37]. The accumulation of multiple, intermittent mountain walking trips along slopes can not only reduce the risk of coronary heart disease but can also improve the physical endurance [35]. In brief, MUGs are likely to permit more health benefits because of the types of intense PA.

Although MUGs have the abovementioned special characteristics to improve public health, substantial empirical studies on this issue have been lacking, so a gap exists between research and practice. Therefore, this study aimed to (1) investigate the effects of MUGs and their surrounding environments on PA in terms of both quality and quantity, (2) identify the key environmental features of MUGs that affect PA, and (3) compare the differences between MUGs and greenways in the plains.

## 2. Methods

### 2.1. Study Area

**Figure 1:**
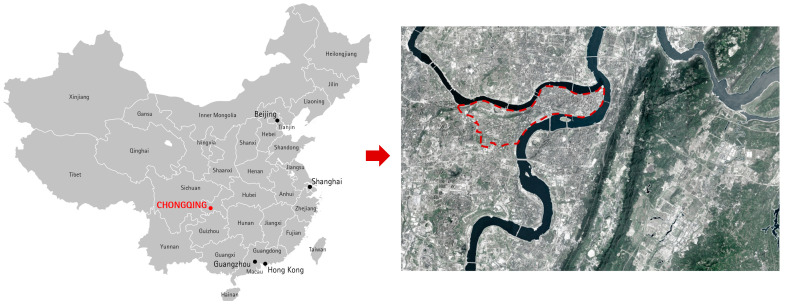
The locations of Chongqing and the Yuzhong Peninsula.

One reason for the scarcity of relevant studies in MUGs is the lack of appropriate samples. The MUG in the Yuzhong Peninsula, Chongqing, China, which is situated in the heart of the city of Chongqing, provides a good case study (Figure 1) because the city is located in a highly mountainous region. The peninsula has a holistic and complicated MUG system, which functions not only as a linear corridor system for ecology but also as part of an urban non-motorized transportation network linking communities and urban clusters. Residents like to travel along this greenway because it connects places separated by the terrain. In some districts, the MUG accounts for 53% of the citizens’ daily travel [33,34]. Since 2010, the municipal government has been expanding and upgrading this MUG, which is the target area of this study.

**Figure 2:**
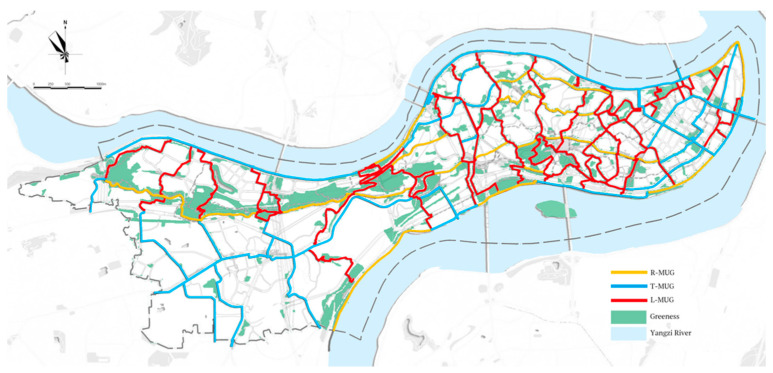
Yuzhong Peninsula MUGs. Source: Chongqing City Bureau of Urban Planning.

The geography of the Yuzhong Peninsula is ‘middle high with surrounding low’ because it is surrounded by the Yangzi and Jialing rivers. We focused on the peninsula’s eastern part because of its mountainous features. The MUG system is structured as a “One Ring–Six Cross–Sixteen Section” area, and its total length is 98.2 km with slopes ranging from 5° to 41°. According to “Planning for the MUG System in Yuzhong District” (Municipal Bureau of Urban Planning, 2017), this system can be categorized into three types: recreational (R-MUG), transportation (T-MUG) and livelihood (L-MUG) (Table 1 and Figure 2). The R-MUG is defined as recreational routes connecting places of interest and providing necessary facilities to support users’ entertainment and recreational activities. It is mostly newly built, whereas the T-MUG and L-MUG have been mainly constructed from the renovation and expansion of an old street system. The T-MUG is supposed to serve as pedestrian-only pathway along the existing road system to connect transit, subways and ferries, which is part of the non-motorized transportation system. The L-MUG mainly serves the communities by connecting the residential buildings to public services and commercial buildings, without necessarily following the current road system. The goal of this project is to provide an urban, linear and green network that serves residents within a 5-min walking distance. Therefore, this study also focused on a buffer area of 300 m along the MUG.

### 2.2. Data

#### 2.2.1. Physical Activity (PA)

With the prevalence of mobile apps and wearable devices, voluntarily geographic information (VGI) technology was developed to obtain mass spatial-behavioral data of users by recording precise details such as location, time, speed and distance [39]. For this study, PA data were obtained from the well-known Chinese sports app KEEP. According to Talking Data (www.Talkingdata.com, accessed on 23 March 2020), the app is very popular and used by people of almost all ages. The average daily shared records have reached 60 million shared entries. The app can record a user’s routes, duration of trips, distances traveled and speed. Users can share and publish their data anytime on social networks such as Weibo and WeChat. The data can be obtained by an API from KEEP and saved in the format of JSON files.

Data collection was conducted in March and September 2019 as the weather is comfortable. The captured VGI data included gender, age, activity duration, distance, speed and path. The information screening exclusion criteria were: (1) Incomplete personal information; (2) The duration was less than 5 min or over 300 min; (3) The route was not all within the scope of the study. Finally, 1314 valid records were obtained and then encoded into ArcGIS to create a dataset. In order to reduce the errors in processing PA and geological data, a 30-m buffer was established where any record that fell within would be counted.

#### 2.2.2. MUG and Surrounding Environment

The MUG data were obtained from official documents: “Yuzhong Peninsula Pedestrian System Planning” and “Planning for MUG System in Yuzhong District” (Municipal Bureau of Urban Planning, 2017). From these documents, the obtained geological data of the MUG were entered into ArcGIS 10.2 (CGCS, 2000).

On the basis of a previous study and the actual characteristics of the MUG in Chongqing, we selected five factors to reflect the surrounding environmental features [27]. The factors were residential density, land-use mixture, transit points of interest (POI), open-space POI, and shops POI [11]. The data for residential density and land-use mixture were obtained from a 2018 land-use map of Chongqing. The data on transit, open-space and shops POI were downloaded from Baidu Maps, which is one of the most popular open-source maps in China. Finally, the data for PA, MUGs and the surrounding environments were superimposed in ArcGIS to form a basic dataset, which was analyzed by ArcToolbox.

### 2.3. Data Analysis

#### 2.3.1. Dependent Variable: Describes Users’ PA in Terms of Both Density and Attributes

(1) PA density: Total PA routes per unit area of the 300 m buffer zone along the MUG, which indicates the density of PA along a certain segment of the MUG. It is calculated by dividing the total length of PA by the buffer area along the route. A blank area would be considered out of use. A total of 1314 records of PA were entered into ArcGIS and processed by the Intersect tool.
(2) PA attributes: Quality of PA, including the duration, distance and speed. Cycling rarely occurs in the Yuzhong Peninsula MUGs, so the types of PA are not counted in this study. These VGI data were obtained from the KEEP app (https://www.keep.com/, accessed on 14 August 2021), then encoded and entered into ArcGIS to be overlaid with the MUG data.

#### 2.3.2. Independent Variables: Describe the Environmental Features of the MUGs

(1) Type ratios: Ranging from 0 to 1, these represent the proportions of the MUG types in a PA record, and are a general reflection of the overall environmental quality. Three ratios correspond to the three MUG types: R-MUG, T-MUG and L-MUG. Each ratio is calculated by dividing the length of the corresponding MUG type by the total length of the PA track records. Features such as pavements, facilities and landscapes have also been considered because each type has its own unified standards.
(2) Slope (elevation-to-distance ratio): Calculated from dividing the elevation by distance in a route and ranges from 0 to 1. It reflects the mountainous characteristics of a MUG. Greenways in the plains tend to have lower slopes.
(3) Node ratio: Nodes are the interactions of greenway segments, which indicates the network feature of a greenway system. Nodes ratios are calculated by dividing the number of nodes by a PA track length in the 300-m buffer zone along each MUG segment. A higher value means better connectivity and reflects the network attributes of MUGs [40].

#### 2.3.3. Control Variables: The Surrounding Environments of the MUGs Could Affect the PA Conducted in Them

Some studies have listed environmental features such as density, diversity, destination, distance and design (5Ds). They were originally defined in a study of transportation behaviors and built environments [27]. For the actual conditions in the Yuzhong Peninsula MUGs, the five factors selected for our study are residential density, land-use mixture, transit POI, open-space POI and shops POI.

We could not obtain accurate sociodemographic information by means of VGI data because many users in the pilot study would hide their real identities. However, KEEP users tend to have similar backgrounds and interests in outdoor activities, so their sociodemographic data were not necessary for the control variables.

### 2.4. Statistical Analysis

Statistical analysis was conducted by SPSS 25.0 (IBM, Armonk, NY, USA). Collinearity diagnostics were performed on the control and independent variables before modeling. The results (VIF < 10) indicated no significant correlations. To explain the relationship between environmental features and PA, we adopted a two-step linear regression model. In the first step, only the control variables were imported to generate Model 1. In the second step, the independent variables were added to generate Model 2. A comparison of the models allowed us to calculate the effect of each independent variable on the dependent variable.

## 3. Results

### 3.1. Descriptive Analysis

#### 3.1.1. PA Density

**Figure 3:**
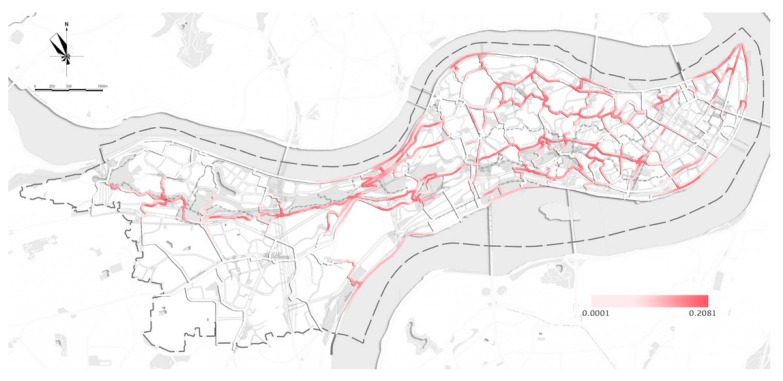
The PA density in the MUG of the Yuzhong Peninsula.

The average MUG density was about 0.44 km/km^2^ with uneven distribution, and more than a half (52.0%) of the MUGs lacked PA records (Table 2). Generally, most PA occurred on east–west-oriented MUGs, whereas only a small proportion occurred on north–south-oriented MUGs (Figure 3). This distribution matched the “middle high with surrounding low” geography of the Yuzhong Peninsula. The PA density of the MUG system was highest in the northeastern and southeastern parts, which are highly developed with high residential density and land-use mixture. The MUGs along the parks, squares, waterfront and transit points showed higher PA density.

**Figure 4:**
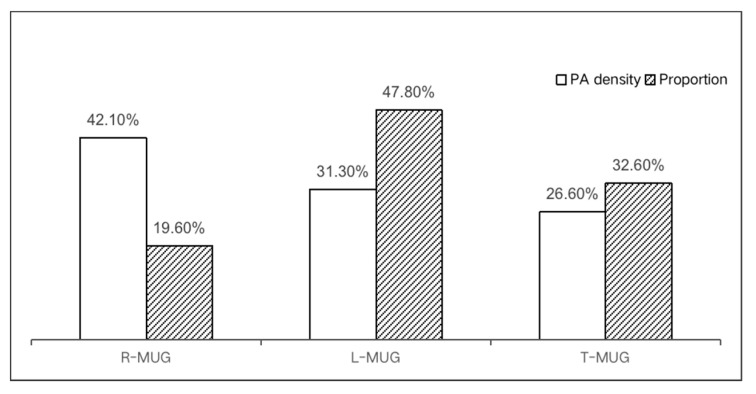
Lengths and PA density proportions of different types of MUGs.

As for PA on the different types of MUGs, R-MUG accounted for 19.6% of the total length but had 42.1% of the PA density with a higher mean value (0.0158). The L-MUG and T-MUG comprised most of the MUG system but accounted for only 57.9% of PA density, with a lower mean value (Table 3) (Figure 4). Therefore, a mismatch occurred between the proportions of the MUGs and the distribution of PA, reflecting the efficiency of the R-MUG in supporting PA density.

#### 3.1.2. PA Attributes

PA attributes consisted of distance, duration and speed, which were also the fundamental factors of PA quality [41]. The mean PA duration was 55.8 min. The R-MUG had the longest duration at about 79.8 min, whereas the T-MUG had the shortest at 32.7 min, indicating that people spent more time on the R-MUG. The mean PA distance was 3.1 km. The average PA distance for the R-MUG was 4.1 km, which was the longest, whereas, for the L-MUG, it was only 2.3 km, indicating that people travel farther on the R-MUG. The mean PA speed was 4.7 km/h. People traveled the fastest at 5.2 km/h on the T-MUG but the slowest at about 3.9 km/h on the L-MUG, indicating that walking was the main type of PA, as the terrain was difficult for cycling and running.

#### 3.1.3. Yuzhong Peninsula MUG and Surrounding Environment

The MUG had a total length of 98.2 km, of which 47.1 km (48%) had records for PA (Figure 2). The density in the northern and eastern parts was higher than in the southern and western parts. The L-MUG and T-MUG are the results of renovation and expansion, so they are strongly associated with the current road system. The R-MUG was mostly built five years ago along an east–west orientation, so it has a slighter slope that is easier for walking.

The mean node ratio was 0.017, which indicates moderate connectivity. The L-MUG had the highest value at 0.021, whereas the R-MUG had the lowest at 0.14, indicating that the former was better connected to the existing road system (Table 4). The mean value of slope was 0.037. The L-MUG was the steepest (0.047), followed by the T-MUG (0.038) and then the R-MUG (0.026) (Table 4).

Within the 300-m buffer zone, the residential density was 31% and land-use mixture was approximately 0.65, indicating that the MUG was located within an area of high urban density and diversity (Table 5). The mean POIs were 8.7 for open-space, 25.4 for transit and 47.9 for shops, indicating that parks and squares were relatively scarce. The L-MUG generally showed the highest values, followed by the R-MUG and T-MUG, indicating that the L-MUG was mostly distributed in a highly urbanized area and had more connections to urban facilities (Table 5).

### 3.2. Statistical Analysis

#### 3.2.1. Model 1. PA Density and MUG Environmental Characteristics

A two-step linear regression method was employed to analyze the relationships between PA density and the environmental features of the MUG. In the first step, control variables representing the features of the surrounding environment were incorporated into the model. Apart from the shops POI, the variables were positively associated with PA density (Table 6). In the second step, independent variables (MUG) were added to the regression. The node, R-MUG and T-MUG ratios were positively associated with PA density, but the slope showed a negative association with it (B = −1.513, *p* < 0.001) and the land-use mixture was not associated. The node ratio showed a slightly positive association (B = 0.009, *p* = 0.021). The step-2 model raised the R-square from 0.113 to 0.264, indicating a better fit with the data even though the land-use mixture was insignificant (Table 6). The analysis showed that both the MUG and its surrounding environment affected PA density.

#### 3.2.2. Model 2. PA Attributes and MUGs Environmental Characteristics

The control variables with the PA attributes of speed, distance and length were imported into the regression. In the sub-model for PA distance, only open-space POI showed a positive association (B = 0.951, *p* < 0.001). For PA duration, residential density (B = 1.156, *p* = 0.014) and PA duration (B = 0.614, *p* < 0.001) were positively associated. For PA speed, only transit POI showed a positive relationship (B = −0.684, *p* < 0.000). The results indicated that the surrounding environment slightly affected these attributes (Table 7).

In the next step, independent variables were imported into the first regression. For PA distance, the open-space POI, R-MUG ratio and node ratio were all positively associated with it, but the association of slope was negative. For PA duration, the same associations were found between these variables. In the second step, the R-square rose from 0.257 to 0.341, signifying that the results of this step had a better fit. Notably, residential density was insignificant, indicating that the MUG itself had a greater influence than did the surrounding environment on PA duration. For speed, the T-MUG ratio was positively associated (B = 0.224, *p* = 0.017), but the slope decreased this (B = −1.546, *p* < 0.001). Transit POI was insignificant in this step. The R-square increased from 0.234 to 0.365, indicating that PA speed was more affected by the MUG than by the surrounding environment. In summary, the results showed that the MUG had stronger effects than did the surrounding environment on the PA attributes (Table 8).

## 4. Discussion

### 4.1. Effects of MUGs and Surrounding Environment on PA Density

In the Yuzhong Peninsula, both the MUG and its surrounding environment affect PA density. This result is consistent with those of previous studies of greenways in the plains [11,20,29,42]. Our study further determined that in mountainous areas, the R-MUG and T-MUG within areas with higher residential density and connectivity to open spaces, as well as with more transit points, were more likely to encourage PA (Table 7). Most of the PA density was distributed in the northeastern part, which is highly urbanized. Hence, increasing the PA density effectively requires matching the MUG to areas with high residential density and more transit points, parks and squares. These findings are similar to those for the plains except that no significant correlation was found for the land-use mixture. Transportation-related PA is usually associated with the land-use mixture [32,43]. In this case, the recreational activities account for a large proportion of the PA because the MUG is a scarce type of recreational space. Cycling and running are prevalent in the plains, which are more closely associated with the land-use mixture. Compared to the slope, the node ratio had less effect, suggesting that the network feature of the MUG did not have as strong an effect as those of the greenways in the plains.

As for the MUG itself, the slope and MUG ratios significantly affected PA density and played fundamental roles in the qualities of the MUG [40]. Users in this mountainous area were more resistant than those living in the plains to walking along steep slopes. The R-MUG ratio showed the most influence on PA density. A possible reason is the better recreational experiences, which are the primary purpose for using the MUG, offered by the R-MUG. Most of the R-MUG is newly built with better standards and slighter slopes. The findings indicate that people in mountainous areas, like those in the plains, prefer greenways with quality, landscapes and natural scenery. This finding is consistent with a previous study that found users would spend time on health, recreational and leisure activities in the greenway [30]. Nature-based recreational experiences and outdoor activities are highly recognized by greenway users [22,28,32].

### 4.2. Effects of of MUGs and Surrounding Environment on PA Attributes

In this case, the MUG itself showed stronger effects than did its surrounding environment on PA attributes. This indicated that people in the mountainous areas, like those living in the plains, prefers to use greenways with better quality. The original purpose of a greenway is to provide the public with a recreational place close to nature [21]. This study indicated that people in the mountainous areas, like those living in the plains, prefer to use greenways with better quality. Unlike the findings of our previous study, the node ratio does not have much effect on the PA attributes and was only slightly associated with PA distance. In the plains, the node ratio represents connectivity, which is closely associated with the accessibility of a greenway [32]. The terrain and complexity of a MUG may render the node ratio an ineffective measure of connectivity in mountainous areas. According to our regression models, the surrounding environment had a very limited influence on the PA attributes. Only open-space POI affected the duration and distance because people may prefer spending more time and walking longer distances in a MUG that is connected to parks and squares. The high residential density and complex terrain in the Yuzhong Peninsula make parks and green spaces more scarce recreational resources, which are more appealing to residents. Like previous studies, residential density showed significant influence on PA density [32,40].

### 4.3. Role of MUGs Classification to Support PA

Policymakers have attempted to classify MUGs in order to regulate their effects on the users’ daily lives. The goal is to improve the quality of urban spaces and landscapes by accurately matching certain types of MUGs with suitable districts. The Yuzhong Peninsula MUG was intended to be accessible and effective at serving the community. According to official guidance, the transformation of existing streets into T-MUGs and L-MUGs is considered to be an economic and effective solution for their accessibility, connectivity, residential density and land-use mixture [27,44]. However, this study contradicts the purpose of the plan by showing that 52.0% of the MUG system is without PA records. Meanwhile, the T-MUG and L-MUG, which constitute 80.4% of the MUG system, accommodate only 57.9% of the total PA density. This low efficiency of supporting PA may be due to a mismatch of the MUG types and their proportions.

Our analysis indicates that the R-MUG is the most effective type in this case for supporting both PA density and attributes because the primary purpose of using this MUG is recreation. This finding is consistent with the findings of previous studies. Connecting users with recreational places is the basic function of greenways [21]. People prefer greenways with high environmental quality for better recreational experiences. In the Yuzhong Peninsula, the R-MUG is equipped with better facilities, scenery, pavements and landscapes with multiple connections to parks, squares and waterfronts. In contrast, the L-MUG and T-MUG are the results of the transformation and renovation of old streets and alleys with few amenities but with more slopes. The role of the R-MUG could also be explained by its average slope, which is the lowest (0.026), and therefore, the most attractive for PA.

Overall, the R-MUG is quite efficient for supporting PA as a result of its better environmental features and slighter slope. However, it only accounts for a small part of the MUG system. This fact hints at classification problems in the current plan for the MUG system.

### 4.4. Key Environmental Feature for Supporting PA in MUG

This study showed some distinct findings from the greenways in the plains in terms of environmental features. The R-MUG, slope and T-MUG were reported to be significantly associated with PA density and attributes. However, the network attributes of greenways are considered to be of great significance to PA in the plains, though the node ratio of the MUG is related only to distance, indicating the weak influence of the network attribute on PA as a possible result of the restrictive terrain. This MUG system has many north–south-oriented greenways, which are not supportive of PA because of the terrain. The R-MUG was found to be significantly supportive of PA, regarding PA density, PA duration and distance. This finding is consistent with the discussion in Section 4.3. In mountainous areas, people prefer recreational activities along MUGs because they are a scarce recreational resource in areas with high urban density such as the Yuzhong Peninsula [32,43]. Another reason is the terrain’s restriction of the construction of more open spaces. As a result, the proportion of recreational activities in the MUG is quite high. Therefore, the recreational qualities of the MUG are essential to PA.

The key features of the surrounding environment in supporting PA are similar to those in the plains. Most of the features are significantly associated with PA density instead of the attributes. For density, residential density, open space, and transit POI show positive relationships. Most PA occurs in the eastern part of the Yuzhong Peninsula because it is more highly developed with dense residential areas, squares and transit points, which have high accessibility and proximity to the MUG. However, the land-use mixture is not associated with PA density in this study. The possible reason is that in the Yuzhong Peninsula, people use the MUG mainly for recreation and not transportation, which is less associated with the land-use mixture. In summary, the surrounding environment shows only a slight association with PA attributes and is focused mostly on open-space POI, indicating that the surrounding environment mainly affects PA density.

### 4.5. Advice to Support PA by Planning in the Practice

The planning for the Yuzhong Peninsula MUG was a top-down process intended to provide residents with multi-purpose linear spaces for recreation, transportation, communication and access to nature. The planners attempted to utilize the classification of MUGs to meet their goal [45]. However, there are no PA records for nearly half of the length of the Yuzhong Peninsula MUG. This is partly because the planning overlooked the scope of daily life and ignored the recreational needs of the residents, leading to lack of use. PA density is unevenly distributed, indicating that the MUG has not fully met the residents’ needs and has been a waste of public resources. A possible reason could be a mismatch between the classification and actual needs. The proportion of the R-MUG in the system is low but people prefer to use it for recreation. The L-MUG and T-MUG are mostly located in developed areas but are of poor quality and have steep slopes.

MUGs do not serve only the ecology but also the wellbeing of residents. Therefore, to improve the efficiency of a MUG in promoting PA, planners and policymakers should concentrate on the match between urban development and the classification of MUGs. The proportions of R-MUG should be increased in areas with certain densities of residence and open spaces, as well as with a number of transit points, so that the R-MUG becomes more accessible and better available to the public. Since most T-MUGs and L-MUGs are already located in highly developed areas, measures should be taken to promote their recreational functions by increasing their environmental qualities, such as better pavement and more amenities and landscapes, while reducing their slopes.

Overall, the match of planning of MUGs with the actual needs of people is the essential problem in this case. Planning could increase the proportion of R-MUGs and enable new MUGs to be installed in densely developed areas. L-MUGs and T-MUGs should be renovated and upgraded with higher standards and slighter slopes to provide better experiences for recreation.

### 4.6. Limitation and Perspective

This study used a VGI method to analyze the environmental features of a MUG and its effects on PA. Although a large amount of data was obtained, some limitations are apparent. These data may not be representative because their socioeconomic information were not fully explored. The mobile app data cannot accurately report the users’ socioeconomic status. As a result, the dynamics behind the relationship of PA and MUGs was not fully explained. Future research should focus on accurate socioeconomic data and intermediary variables. Data from questionnaires could be combined with observed data. Moreover, as a case study, this article cannot accommodate all the mountainous and urban settings due to the data limit. MUG systems can be classified into different types. The range and Applicability of the findings need to be further tested in a global scope.

Finally, An international perspective into comparing the prevalence of PA for populations in the different topographic setting could be helpful to understand how the topography influences PA as environmental attributes (Appendix A Table A1).

## 5. Conclusions

This study examined the effects of the environmental features of a MUG by means of a case study to identify the key features that might influence PA density and attributes. Both a MUG and its surrounding environment could affect PA density, which is generally consistent with the findings of previous studies. Compared to MUG-surrounding environments, in this case, PA attributes were more affected by MUG environmental features including the slope, R-MUG and T-MUG ratio. Overall, these findings contribute as the first step toward investigating MUGs and add to existing knowledge on urban greenways and PA. They also provide evidence and suggestions for improving MUGs so that they encourage PA.

This study applied a VGI method to collect objective PA data, which was able to process the data with ease and determine the PA distribution on the greenway. The reliability and accuracy of this method are relatively high on a larger scale and demonstrate an efficient approach to future environmental and behavioral studies.

The main conclusions are that: (1) Similar to urban greenways in plains, both MUGs and their surrounding environments can affect PA density; (2) In this case, MUGs affect PA attributes more than do their surrounding environments, which are different from those in the plains; (3) R-MUG is the most supportive type of MUG for PA in the Yuzhong Peninsula and recreation is the primary purpose for people to use MUGs.

A MUG is not the same as an urban greenway in the plains in terms of encouraging PA. To better promote PA, the special environmental features of MUGs should be considered instead of just applying existing experiences from plains. Recently, many greenway projects have been ongoing in China, especially in mountainous areas. In this study, the potential of the Yuzhong Peninsula MUG to encourage PA was not fully explored because of the mismatch between planning and users’ actual needs. The classification of MUGs should be reassessed to meet people’s actual demands. Environmental qualities and the degrees of the slopes should be considered in planning. Future studies should target specific aspects of MUGs. Compared to ecological, cultural and other functions, the roles of MUGs in serving the wellbeing of residents have not yet been fully explored. More consideration of PA should be incorporated into the planning of MUGs so that they contribute to more balanced public health and built environments in mountainous areas.

## Figures and Tables

**Table 1:** Classification of Yuzhong Peninsula MUGs. Original Source: Chongqing City Bureau of Urban Planning.

MUG Type	Description	Source	Proportion	Photo
Transportation (T-MUG)	Non-motor pathway connects transit to urban clusters and provides people routes for traveling.	Mainly from the renovation of walkways.	33.4% (32.80 km)	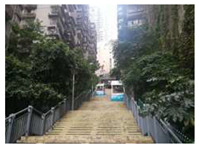
Recreational (R-MUG)	Walkways connect attractions and places of interest to communities, providing people places for hiking, sightseeing and other types of recreational activities.	Newly built greenway.	19.6% (19.20 km)	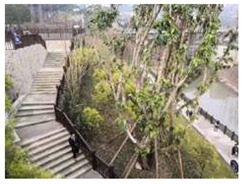
Livelihood (L-MUG)	Pedestrian-only pathways connect communities and public facilities, providing people places for shopping, socializing and visiting.	Mainly from conservation and extension of existing streets.	47.0% (46.20 km)	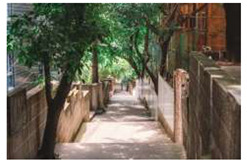

**Table 2:** The MUG with the presence of PA.

	R-MUG (km)	T-MUG (km)	L-MUG (km)	Total (km)
PA presence	14.8	13.5	18.8	47.1 (48.0%)
No presence	4.4	19.3	27.4	51.1 (52.0%)
Total	19.2 (19.6%)	32.8 (33.4%)	46.2 (47.0%)	98.2 (100.0%)

**Table 3:** PA density and attributes.

Type	PA Density (km/km^2^)		PA Attributes					
			Duration (min)		Distance (km)		Speed (km/h)	
	Range	Mean	Range	Mean	Range	Mean	Range	Mean
R-MUG	0.0020–0.1728	0.0158	30.0–424.6	79.8	1.4–12.1	4.1	2.1–6.4	5.0
T-MUG	0.0001–0.2081	0.0072	20.2–104.3	32.7	1.1–15.2	2.9	1.8–7.3	5.2
L-MUG	0.0001–0.1370	0.0048	27.2–217.2	54.9	1.0–13.8	2.3	1.9–5.4	3.9

**Table 4:** Environmental features of the MUG.

Type	Slope		Node Ratio (n/km)	
MUG	Range	Mean	Range	Mean
R-MUG	0.006–0.079	0.026	0.002–0.034	0.014
T-MUG	0.012–0.141	0.038	0.006–0.051	0.016
L-MUG	0.019–0.189	0.047	0.004–0.079	0.021

**Table 5:** Environmental features of surroundings.

Type	Residential Density (%)		Mixture of Land Use		Open Space POI (n/km)		Transit POI (n/km)		Shop POI (n/km)	
	Range	Mean	Range	Mean	Range	Mean	Range	Mean	Range	Mean
R-MUG	6–29	18	0.07–0.82	0.57	4–24	12.5	7–29	18.4	19–48	32.5
T-MUG	15–45	27	0.16–0.85	0.71	1–9	7.8	15–48	35.2	25–64	45.1
L-MUG	21–69	48	0.37–0.87	0.67	0–10	5.9	11–30	22.5	38–89	66.2

**Table 6:** Linear regression model of PA density.

Model 1	Factor	1st StepB *p*-Value		2nd StepB *p*-Value	
Control variable (Surrounding environmental characteristic of MUG)	Residential density	**2.145** *	0.000	**2.674** *	0.000
	Mixture of land use	**3.121** *	0.030	7.441	0.312
	Open space POI	**1.152** *	0.000	**2.32** *	0.000
	Transit POI	**5.218 ** *	0.021	**1.239 ** *	0.001
	Shop POI	0.021	0.821	0.002	0.119
Independent variable (MUG characteristics)	Node ratio			**0.009** *	0.021
	Slope			**−1.513** *	0.000
	T-MUG ratio			**1.531 ** *	0.002
	L-MUG ratio			0.625	0.127
	R-MUG ratio			**2.314** *	0.000
Constant		−0.167	0.219	−0.128	0.651
R square		0.113		0.264	

Note: * *p* < 0.05. Sample size = 1314. B = standardized beta. Bold data means the value is statistically significant.

**Table 7:** Linear regression model of PA attributes: Step 1.

Model 2: Step 1	Factor	PA Attribute		Duration		Speed	
		Distance					
		B	Sig	B	*p*-Value	B	Sig
Control variable (Surrounding environmental characteristic of MUG)	Residential density	−0.027	0.346	**1.156** *	0.014	−6.514	0.540
	Mixture of land use	−4.581	0.453	−2.841	0.515	−0.358	0.815
	Open space POI	**0.951** *	0.000	**0.614** *	0.001	0.935	1.548
	Transit POI	0.622	0.356	0.561	0.096	**−0.684** *	0.000
	Shop POI	4.156	0.678	2.159	0.156	3.256	0.681
Constant			0.053		0.078		0.652
R square		0.257		0.234		0.201	

Notes: * *p* < 0.05. Sample size = 1314. B = standardized beta. Bold data means the value is statistically significant.

**Table 8:** Linear regression model of PA attributes: Step 2.

Model 2: Step 1	Factor	PA Attribute		Duration		Speed	
		Distance					
		B	*p*-Value	B	*p*-Value	B	*p*-Value
Control variable (Surrounding environmental characteristic of a MUG)	Residential density	−0.944	0.346	0.035	0.531	−0.195	0.062
	Mixture of land use	−0.751	0.453	−0.197	0.680	−0.130	0.256
	Open space POI	**0.219** *	0.000	**0.192** *	0.000	0.638	0.144
	Transit POI	0.169	0.626	0.175	0.096	−0.195	1.210
	Shop POI	0.097	0.090	0.201	0.156	0.523	0.129
Independent variable (MUG characteristics)	Node ratio	**0.142** *	0.013	0.223	0.087	0.147	0.982
	Slope	**−0.741** *	0.006	−0.516	0.617	**−1.546** *	0.000
	T-MUG ratio	0.089	0.052	**0.340** *	0.027	**0.224** *	0.017
	L-MUG ratio	−0.055	0.414	0.190	0.247	−0.008	0.994
	R-MUG ratio	**0.323** *	0.000	**1.273** *	0.000	0.015	0.114
Constant			0.005		0.021		0.681
R square		0.341		0.365		0.282	

Notes: * *p* < 0.05. Sample size = 1314. B = standardized beta. Bold data means the value is statistically significant.

## Data Availability

The data that support the findings of this study are available on reasonable request from the corresponding author M.M. The data are not publicly available due to the agreement of privacy protection with the data provider.

## Appendix A

**Table A1:** The descriptive analysis of MVPA from different environmental settings across 10 countries of the world (modified from Sallis et al., 2016 [12]).

	All Cities	Ghent, Belgium	Curitiba, Brazil	Bogota, Colombia	Olomouc, Czech Republic *	Aarhus, Denmark	Hong Kong, China ^†^	Cuernavaca, Mexico *	Wellington, New Zealand *	Christchurch, New Zealand *	Stoke- on-Trent, UK	Seattle, WA, USA *	Baltimore, MD, USA *
Number with ≥4 days of valid physical activity data	6822 (68%)	1050 (90%)	330 (47%)	223 (23%)	258 (78%)	272 (42%)	269 (56%)	656 (97%)	416 (84%)	373 (75%)	135 (16%)	1198 (93%)	870 (95%)
MVPA (min/day)	37·3 (26·5)	35·5 (23·5)	31·5 (24·6)	37·0 (26·4)	47·1 (27·7)	39·7 (23·2)	44·9 (25·3)	31·2 (25·2)	50·1 (31·0)	44·0 (32·5)	36·7 (27·3)	36·3 (24·9)	29·2 (22·0)

Data are mean (SD) or *n* (%) unless otherwise indicated. Totals might not be exactly 100% because of rounding. MVPA = moderate to vigorous physical activity. * Study city aimed to collect accelerometer data in the total sample. ^†^ Study city aimed to collect accelerometer data in a fixed proportion of the total sample.
